# The COVID-19 ambulance response assessment (CARA) study: a national survey of ambulance service healthcare professionals’ preparedness and response to the COVID-19 pandemic

**DOI:** 10.29045/14784726.2024.3.8.4.10

**Published:** 2024-03-01

**Authors:** Jack William Barrett, Kate Bennett Eastley, Anthony Herbland, Peter Owen, Salman Naeem, Craig Mortimer, James King, Theresa Foster, Nigel Rees, Andy Rosser, Sarah Black, Fiona Bell, Rachael Fothergill, Adam Mellett-Smith, Michelle Jackson, Graham McClelland, Paul Gowens, Robert Spaight, Sandra Igbodo, Martina Brown, Julia Williams

**Affiliations:** South East Coast Ambulance Service NHS Foundation Trust ORCID iD: https://orcid.org/0000-0002-0040-537X; University of Surrey ORCID iD: https://orcid.org/0000-0002-2358-4497; University of Hertfordshire ORCID iD: https://orcid.org/0000-0001-6182-4191; South East Coast Ambulance Service NHS Foundation Trust; Barts Health NHS Trust ORCID iD: https://orcid.org/0000-0002-0153-1669; South East Coast Ambulance Service NHS Foundation Trust ORCID iD: https://orcid.org/0000-0001-6989-2244; University of Hertfordshire ORCID iD: https://orcid.org/0000-0001-9259-0957; East of England Ambulance Service NHS Trust ORCID iD: https://orcid.org/0000-0002-6395-0885; Welsh Ambulance Services NHS Trust ORCID iD: https://orcid.org/0000-0001-8799-5335; West Midlands Ambulance Service University NHS Foundation Trust ORCID iD: https://orcid.org/0000-0002-5477-4269; South Western Ambulance Service NHS Foundation Trust ORCID iD: https://orcid.org/0000-0001-6678-7502; Yorkshire Ambulance Service NHS Trust ORCID iD: https://orcid.org/0000-0003-4503-1903; London Ambulance Service NHS Trust ORCID iD: https://orcid.org/0000-0003-1341-6200; London Ambulance Service NHS Trust ORCID iD: https://orcid.org/0000-0002-6157-8979; North East Ambulance Service NHS Foundation Trust; North East Ambulance Service NHS Foundation Trust ORCID iD: https://orcid.org/0000-0002-4502-5821; Scottish Ambulance Service ORCID iD: https://orcid.org/0000-0002-9311-3885; East Midlands Ambulance Service NHS Trust ORCID iD: https://orcid.org/0000-0003-4361-5876; North West Ambulance Service NHS Trust ORCID iD: https://orcid.org/0009-0001-8290-0912; South Central Ambulance Service NHS Foundation Trust ORCID iD: https://orcid.org/0000-0003-3083-8958; South East Coast Ambulance Service NHS Foundation Trust; University of Hertfordshire; College of Paramedics ORCID iD: https://orcid.org/0000-0003-0796-5465

**Keywords:** COVID-19, mental health, occupational health, staff well-being

## Abstract

**Background:**

The COVID-19 pandemic placed significant demand on the NHS, including ambulance services, but it is unclear how this affected ambulance service staff and paramedics in other clinical settings (e.g. urgent and primary care, armed services, prisons). This study aimed to measure the self-perceived preparedness and impact of the first wave of the pandemic on paramedics’ psychological stress and perceived ability to deliver care.

**Methods:**

Ambulance clinicians and paramedics working in other healthcare settings were invited to participate in a three-phase sequential online survey during the acceleration (April 2020), peak (May 2020) and deceleration (September/October 2020) phases of the first wave of COVID-19 in the United Kingdom. Recruitment used social media, Trust internal bulletins and the College of Paramedics’ communication channels, employing a convenience sampling strategy. Data were collected using purposively developed open- and closed-ended questions and the validated general health questionnaire-12 (GHQ-12). Data were analysed using multi-level linear and logistic regression models.

**Results:**

Phase 1 recruited 3717 participants, reducing to 2709 (73%) by phase 2 and 2159 (58%) by phase 3. Participants were mostly male (58%, n = 2148) and registered paramedics (n = 1992, 54%). Mean (standard deviation) GHQ-12 scores were 16.5 (5.2) during phase 1, reducing to 15.2 (6.7) by phase 3. A total of 84% of participants (n = 3112) had a GHQ-12 score ≥ 12 during the first phase, indicating psychological distress. Participants that had higher GHQ-12 scores were feeling unprepared for the pandemic, and reported a lack of confidence in using personal protective equipment and managing cardiac arrests in confirmed or suspected COVID-19 patients.

**Conclusions:**

Most participants reported psychological distress, the reasons for which are multi-factorial. Ambulance managers need to be aware of the risks to staff mental health and take action to mitigate these, to support staff in the delivery of unscheduled, emergency and urgent care under these additional pressures.

## Background

COVID-19 was declared a pandemic by the World Health Organization on 11 March 2020. The pandemic placed considerable demand on healthcare services across the world, including the NHS (Berger et al., 2022; McCabe et al., 2020; Verelst et al., 2020; Winkelmann et al., 2022). Ambulance services in the United Kingdom were responsible for telephone triage, direct patient assessment and transporting suspected COVID-19 cases. This patient interaction occurred in unfamiliar and dynamically changing environments, with clinicians further constrained by wearing additional personal protective equipment (PPE) and assimilating frequently changing guidance from national and local bodies.

The health and well-being of paramedics has gained considerable attention over the last 20 years, due to a multitude of factors: sick leave absence is higher in ambulance staff than in other areas of the NHS (Taylor et al., 2022), operational and organisational demands placed on the ambulance workforce (Clark et al., 2021), increasing demand on ambulance services to tackle urgent, rather than emergency, care needs and the absence of managerial support (Clark et al., 2021; Lawn et al., 2020; Taylor et al., 2022). COVID-19 compounded these issues further, and while it is difficult ascertain the cost of life and long-term health implications related to being a healthcare worker during COVID-19, it has had an impact on the resilience and morale of staff (Poon et al., 2022; Taylor et al., 2022).

Quantifying the impact COVID-19 had on ambulance staff in the United Kingdom is challenging; one qualitative study suggested that Welsh paramedics held genuine concerns for their personal safety and that of their families, and about the availability and accessibility of PPE and the impact of the pandemic on their clinical decision making (Rees et al., 2021). Evidence from other healthcare systems suggests that COVID-19 was associated with negatively impacting the mental health of ambulance staff (Blanchard et al., 2022; Ebben et al., 2023; Monteiro Fonseca et al., 2021; Vujanovic et al., 2021).

A UK-wide quantitative study of emergency department (ED) doctors highlighted the impact that working during the pandemic had on physical and psychological well-being (Roberts et al., 2021). Paradoxically, studies from previous pandemics found that psychological effects experienced by healthcare professionals did not differ from the general population, likely due to training, experience and confidence in infection and prevention control procedures (Chua et al., 2004; Lancee et al., 2008).

This study aimed to understand the evolving and cumulative effects of working during the COVID-19 pandemic on the psychological health of ambulance service staff and paramedics employed in other healthcare settings.

## Methods

### Study design, setting and participants

The COVID-19 ambulance response assessment (CARA) study was a prospective online three-phase sequential survey of paramedics and ambulance clinicians in the United Kingdom. Eligible participants included ambulance service staff involved in patient contact, either face to face or via telephone, plus paramedics working in other settings (e.g. urgent and primary care, armed services, prisons). This article focused on the quantitative aspect of the study; findings from the qualitative component can be found in Eaton-Williams and Williams (2023).

The survey was advertised through the College of Paramedics and participating NHS ambulance trusts’ media platforms (i.e. emails, news bulletins, social media), and employed a convenience sampling strategy. It was hosted via the platform ‘online surveys’, which is fully compliant with Good Clinical Practice, 21 CFR Part 11, GDPR, ISO 27001, including stringent data security procedures and private servers.

Survey data collection took place in three phases during the first wave of the COVID-19 pandemic in the United Kingdom. Phase 1 was during the acceleration of the COVID-19 pandemic (2–15 April 2020), phase 2 during the peak (2–13 May 2020) and phase 3 during the deceleration of the first wave (21 September–12 October 2020). These dates were chosen through consensus by the study team based on the published data of COVID-19 cases and deaths available at the time.

Participants who completed phase 1 and wished to continue were asked to provide a contact email address; those who complied were later contacted for subsequent phases, with eligibility for phase 3 dependent on completing phase 2 and overall eligibility hinging on completing phase 1 of the survey.

### Survey measures

The study team designed the survey questions and piloted them with a group of experienced paramedic researchers to ensure face validity. The survey included closed- and open-ended questions (Supplementary 1) addressing participants’ demographics, PPE availability and training and perceived confidence in using PPE, managing out-of-hospital cardiac arrests and performing aerosol-generating procedures (AGPs), including, for example:

*28. What is your response to the following statement: I feel confident about managing cardiac arrest in suspected COVID-19 patients?*

*29. How many times have you experienced any absence of PPE when involved in direct clinical contact with suspected COVID-19 cases?*

To assess participants’ stress response, the general health questionnaire-12 (GHQ-12) (Goldberg & Hillier, 1979) was used following the approach taken in a similar study with ED doctors (Roberts et al., 2021). The GHQ-12 includes 12 statements using a four-point Likert scale (0 most positive, 3 most negative), generating a total score ranging from 0 to 36; a score ≥ 12 is defined in the literature as at risk of psychological distress (Goldberg & Williams, 1988).

### Statistical analysis

Descriptive statistics were used to describe participant flow through each phase and presented as counts (percentages) for categorical variables and means (standard deviations) and medians (inter-quartile ranges) for continuous variables. Analyses were conducted using StataCorp 2019 (Stata statistical software: release 16, College Station, TX: StataCorp LLC).

Variables were categorised retrospectively to reduce the number of categories for the modelling process: age (categorised to decades), primary role (patient-facing, remote patient-facing and other) and ethnicity (white, minoritised ethnic group and ‘prefer not to say’). The research team grouped predictors believed to be associated with GHQ-12 scores into:

1. demographic factors (age, sex, ethnicity, role);
2. preparedness to deal with a global pandemic (confidence in the availability and use of PPE, general feelings of preparedness, experience of working during previous infectious disease outbreaks, confidence in treating COVID-19 patients in cardiac arrest;
3. current state of health (presence/absence of a physical and mental health condition); and
4. source/frequency of COVID-19 information.

There were no missing data for GHQ-12 scores, demographic data or health status (physical and mental health conditions), as these were required fields. Complete case analysis was used to handle missing data.

A series of linear regression models were fitted to the data at each phase to examine the factors that may have impacted GHQ-12 scores. After fitting the mean (unadjusted) model, groups of predictors, as described above, were added to the model to produce a succession of nested models. For each model, we report the adjusted estimates and 95% confidence intervals (CIs) of the GHQ-12 score. Multi-level linear regression models were then fitted to the data, which included each survey phase as categorical fixed effects and the predictors described above, including random subject effects to account for the correlation between multiple observations from the same individual. Predictor variables were not included in the mixed effect models if they were unavailable at all three time points. The multi-level model, which captures the data from all three phases, is the final reported model included in the main results.

At each survey phase, Cronbach’s alpha for GHQ-12 was acceptable, with scores of 0.82, 0.85 and 0.92, respectively. To further explore predictors for being ‘at risk’ of suffering from psychological distress (i.e. GHQ-12 score ≥ 12), we fitted a series of multi-level logistic regression models with ‘at risk’ as a binary variable. The estimated effects were reported as odds ratios, with an odds ratio of ≤ 1 indicating that the odds of being ‘at risk’ are lower in that group compared to the reference group.

A formal power calculation was not performed due to the difficulty in getting a definitive number on the true size of the population of clinicians working in the ambulance service and paramedics working outside the ambulance service.

## Results

### Participant demographics

Phase 1 recruited 3717 participants, reducing to 2709 (73%) by phase 2 and 2159 (58%) by phase 3 (Table 1). Males represented over half of the participants, and this proportion persisted throughout each phase (n = 2148, 58%; n = 1571, 58%; n = 1270, 59%; respectively). Paramedic was the most common professional role (n = 1992, 54%); the majority had been registered for more than two years (n = 1963, 53%). Most participants (70%) had not worked in a clinical role during a previous infectious disease outbreak and reported being in a patient-facing role (n = 3055, 82%), and only 331 (9%) had been redeployed from their usual role to a front-line role.

**Table 1.** Participant demographics.

		Phase 1	Phase 2	Phase 3
	**Category**	N = 3717	N = 2709	N = 2159
		n (%)	n (%)	n (%)
**SEX**	Female	1551 (42)	1126 (42)	880 (41)
	Male	2148 (58)	1571 (58)	1270 (59)
	Prefer not to say	18 (< 0.5)	12 (< 0.5)	9 (< 0.5)
**AGE**	< 20	37 (1)	29 (1)	20 (1)
	21–25	418 (11)	298 (11)	221 (10)
	26–30	669 (18)	453 (17)	341 (16)
	31–35	605 (16)	440 (16)	347 (16)
	36– 40	443 (12)	330 (12)	276 (13)
	41– 45	496 (13)	374 (14)	296 (14)
	46–50	483 (13)	358 (13)	303 (14)
	51–55	321 (9)	240 (9)	195 (9)
	56–60	197 (5)	149 (6)	127 (6)
	61–65	42 (1)	35 (1)	30 (1)
	66–70	5 (< 1)	3 (< 1)	3 (< 1)
	> 70	1 (< 1)	0	0
**PROFESSIONAL ROLE**	Paramedic	1992 (54)	*1501 (55)*	1232 (57)
	Advanced technician / technician	284 (8)	187 (7)	130 (6)
	Emergency care assistant	253 (7)	162 (6)	127 (6)
	Specialist paramedic	239 (6)	189 (7)	158 (7)
	Other	197 (5)	138 (5)	103 (5)
	Student paramedic	164 (4)	118 (4)	86 (4)
	Advanced paramedic	156 (4)	114 (4)	90 (4)
	Associate ambulance practitioner	139 (4)	99 (4)	79 (4)
	HART/SORT	84 (2)	55 (2)	41 (2)
	Call handler	72 (2)	48 (2)	35 (2)
	Emergency medical advisor	63 (2)	41 (2)	30 (1)
	Telephone clinical advice	40 (1)	33 (1)	29 (1)
	Nurse	22 (< 1)	15 (< 1)	13 (< 1)
	Consultant paramedic	12 (< 1)	9 (< 1)	6 (< 1)
LENGTH OF REGISTRATION, IF REGISTERED*	< 12 months	350 (13)	256 (13)	*N/A*
	1–2 years	300 (11)	226 (12)	*N/A*
	2–5 years	584 (22)	*413 (21)*	*N/A*
	5–10 years	579 (22)	439 (16)	*N/A*
	10–15 years	404 (15)	321 (16)	*N/A*
	> 15 years	396 (15)	304 (15)	*N/A*
	Not registered	1104 (30)	750 (28)	*N/A*
PRIMARY ROLE SINCE COVID-19*	PF – physical contact clinical care	3055 (82)	*N/A*	*N/A*
	PF – remote contact (e.g. call centre or clinical advice)	273 (7)	*N/A*	*N/A*
	Managerial	186 (5)	*N/A*	*N/A*
	COVID-19 hub	24 (< 1)	*N/A*	*N/A*
	Research	19 (< 1)	*N/A*	*N/A*
	Support	28 (< 1)	*N/A*	*N/A*
	Training	72 (2)	*N/A*	*N/A*
	Other	60 (2)	*N/A*	*N/A*
**REDEPLOYED**	Yes	331 (9)	*N/A*	*N/A*
	No	3386 (91)	*N/A*	*N/A*
PREVIOUS INFECTIOUS DISEASE OUTBREAKS**	Yes	1105 (30)	*N/A*	*N/A*
	No	2612 (70)	*N/A*	*N/A*

*Data were not collected during phase 3.

**These included influenza, Ebola, cholera, severe acute respiratory syndrome, Middle East respiratory syndrome and coronavirus. Participants were able to select which they had experienced; given the low individual responses, these have been amalgamated.

HART: hazardous area response team; PF: patient-facing; SORT: special operations response team.

### Personal health and COVID-19

A similar proportion of pre-existing physical or mental health conditions was reported (n = 1347, 36%; n = 1401, 38%; respectively) (Supplementary 2). The majority of these participants were concerned that exposure to COVID-19 would worsen their pre-existing physical or mental health conditions (n = 1048, 78%). While most saw no change in their physical health (n = 686, 66%), participants with a pre-existing mental health condition reported their condition worsened between phase 1 and phase 2 (n = 630, 58%). The easing of lockdown was reported to not have influenced a change in most participants with a physical health condition (n = 686, 73%), whereas only half of participants reported no change in their mental health condition (n = 460, 49%).

Most participants agreed that their personal health was at risk during phase 1 (n = 3056, 82%), but reported that their health stayed the same during the phase 2 and 3 surveys (n = 1486, 55%; n = 1207, 56%; respectively). However, there was a group of participants whose health got worse due to their clinical role through phases 2 and 3 (n = 1172, 42%; n = 773, 32%; respectively). The perceived risk of becoming infected by COVID-19 remained a worry for most participants throughout all phases (n = 2961, 80%; n = 1898, 70%; n = 1256, 58%; respectively).

### Self-reported confidence

Participants were polarised in their self-reported confidence in using PPE during phase 1, as either being very confident (n = 1139, 31%) or unconfident (n = 1570, 42%); however, by phase 3, only 431 (20%) participants reported they were not confident in using PPE (Table 2). During phase 1, most participants reported they were not confident in managing patients with suspected COVID-19 who were in cardiac arrest (n = 1836, 56%). This proportion had decreased by phase 2 (n = 891, 37%). In phase 3, participants were not asked about their confidence level, but whether their confidence had changed throughout the study. The majority reported it had not (n = 770, 40%), while relatively equal proportions reported their confidence as either improved (n = 426, 22%) or worse (n = 306, 16%). Confidence in performing AGPs and caring for patients with COVID-19 showed a similar trend, with participant confidence polarised during phase 2 but more balanced by phase 3.

**Table 2.** Participant self-reported confidence in using PPE, managing patients in cardiac arrest with suspected COVID-19, carrying out AGPs and providing care to patients with COVID-19.

		Phase 1	Phase 2	Phase 3
		N = 3717	N = 2709	N = 2159
		n (%)	n (%)	n (%)
**CONFIDENCE IN USING PPE**	Very confident	1065 (29)	172 (6)	205 (10)
	Somewhat confident	74 (2)	850 (31)	841 (39)
	Neither	612 (16)	574 (21)	452 (21)
	Somewhat unconfident	474 (13)	578 (21)	278 (13)
	Very unconfident	1096 (29)	249 (9)	153 (7)
	Missing	396 (11)	286 (11)	220 (10)
**CONFIDENCE IN MANAGING CARDIAC ARREST IN SUSPECTED COVID-19 PATIENTS**	Very confident	167 (5.0)	223 (9.2)	N/A
	Somewhat confident	697 (21)	808 (33)	N/A
	Neither	617 (19)	501 (21)	N/A
	Somewhat unconfident	1050 (32)	608 (25)	N/A
	Very unconfident	786 (24)	283 (12)	N/A
	Always confident	N/A	N/A	342 (18)
	Improved	N/A	N/A	426 (22)
	No change	N/A	N/A	770 (40)
	Got worse	N/A	N/A	306 (16)
	Never confident	N/A	N/A	95 (4.9)
**CONFIDENCE IN CARRYING OUT AGP**	Very confident	N/A	189 (7.8)	370 (19)
	Somewhat confident	N/A	728 (30)	348 (18)
	Neither	N/A	461 (19)	861 (45)
	Somewhat unconfident	N/A	645 (27)	214 (11)
	Very unconfident	N/A	400 (17)	133 (6.9)
**HOW PREPARED DID YOU FEEL TO PROVIDE DIRECT CARE TO PATIENTS WHO WERE SUSPECTED OR CONFIRMED TO BE COVID-19 POSITIVE?**	Very prepared	187 (5.0)	247 (9.1)	281 (13)
	Somewhat prepared	1131 (30)	1044 (39)	898 (42)
	Neither prepared nor unprepared	469 (13)	465 (17)	485 (22)
	Somewhat unprepared	1501 (40)	770 (28)	409 (19)
	Completely unprepared	429 (12)	183 (6.8)	86 (4)

AGP: aerosol-generating procedure; PPE: personal protective equipment.

Most participants received formal instructional videos, written instruction or simulation training (n = 2100, 63%). In most cases, they reported that they never experienced a lack of PPE (Supplementary 3). When PPE was reported absent, it was seen most frequently during phase 1 and reduced through phases 2 and 3.

### General health questionnaire-12

**Figure 1:**
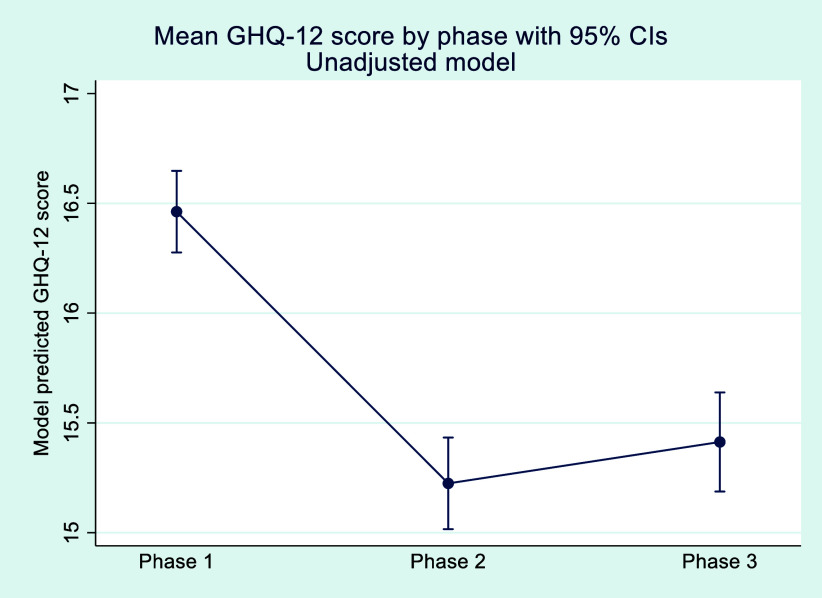
Figure 1. Mean GHQ-12 scores through each phase of the study.

Overall, mean GHQ-12 scores were 16.5 (± 5.2) during phase 1, reducing to 15.2 (± 6.7) by phase 3 (Figure 1). Females had a higher mean GHQ-12 score than males (17.1 (± 5.1) vs 16 (± 5.2), p < 0.001). However, both male and female participants saw a decline in their mean scores. While the difference between the sexes remained significant at phase 2 (p < 0.001), no significant difference was observed in phase 3 (15.5 (± 6.6) vs 15.0 (± 6.7), p = 0.08).

Mean scores (SD) were 16.5 (5.2), 15.1 (8.9) and 15.2 (6.7), respectively.

CI: confidence interval; GHQ-12: general health questionnaire-12; SD: standard deviation.

The proportion of participants at risk of psychological distress (GHQ-12 ≥ 12) was highest in phase 1 (n = 3112, 84%) and reduced throughout the study (n = 1897, 70%; n = 1439, 67%; respectively). More females than males were at risk of psychological distress in all phases (Table 3; Supplementary 4).

**Table 3.** Summary table of participants at risk of psychological distress.

GHQ-12 score			Phase 1	Phase 2	Phase 3
			3717	2709	2159
	**Likert scale (0-1-2-3)**				
**ALL**		n (%) ≥ 12	3112 (84)	1897 (70.0)	1439 (66.7)
**SEX**	Female	n (%) ≥ 12	1367 (88)	858 (76)	618 (70)
	Male	n (%) ≥ 12	1728 (80)	1031 (66)	816 (64)
	Prefer not to say	n (%) ≥ 12	17 (94)	8 (67)	5 (56)
**PRIMARY ROLE**	PF – direct	n (%) ≥ 12	2571 (84)	1573 (70)	1184 (66)
	PF – remote	n (%) ≥ 12	238 (87)	143 (75)	100 (66)
	Other	n (%) ≥ 12	303 (78)	181 (65)	155 (70)
**REDEPLOYED**	Yes	n (%) ≥ 12	274 (83)	190 (74)	150 (70)
	No	n (%) ≥ 12	2838 (84)	1707 (70)	1289 (66)
PREVIOUS INFECTIOUS DISEASE OUTBREAKS*	Yes	n (%) ≥ 12	923 (84)	572 (71)	473 (70)
	No	n (%) ≥ 12	2189 (84)	1325 (70)	966 (65)

*These included influenza, Ebola, cholera, SARS, MERS-CoV and others.

GHQ-12: general health questionnaire-12; PF: patient-facing.

Confidence in using PPE was a strong predictor in GHQ-12, with being confident predicting a lower GHQ-12 score (0.9, 95% CI 0.41 to 1.39, p < 0.001) and being very unconfident predicting a higher score (3.88, 95% CI 3.30 to 4.47, p < 0.001). Other factors that predicted a higher GHQ-12 score were pre-existing mental health conditions (1.6, 95% CI 1.33 to 1.87, p < 0.001), clinical experience of previous infectious disease outbreaks (0.64, 95% CI 0.31 to 0.98, p < 0.001) and feeling unprepared for the pandemic (2.15, 95% CI 1.66 to 2.65, p < 0.001). Meanwhile, being male predicted lower GHQ-12 scores (-0.91, 95% CI -1.22 to -0.60, p < 0.001) and accessing Trusts’ guidance monthly compared with hourly (-1.44, 95% CI -2.43 to -0.45, p = 0.004) was predictive of lower GHQ-12 scores.

## Discussion

This survey found that 84% of participants experienced psychological distress as measured by the GHQ-12. While mean GHQ-12 scores decreased over the study (16.5 ± 5.2–15.2 ± 6.7), they remained higher than the general population (16.5 vs 11.6) (Davillas & Jones, 2021) and ED doctors (16.5 vs 13.0) (Roberts et al., 2021) recorded over the same period. Our analysis suggests that higher GHQ-12 scores were associated with low confidence in using PPE, feeling unprepared, having pre-existing mental health conditions and a higher frequency of accessing COVID-19 guidance.

The provision of PPE received widespread media attention and criticism during the outbreak of COVID-19 due to supply chain issues that placed front-line healthcare professionals at risk of contracting the virus and were perceived to contribute to doctors’ psychological distress (McKee, 2020; Ranney et al., 2020; Roberts et al., 2021; Tabah et al., 2020). Paramedics expressed concerns about the appropriateness of the PPE available and that PPE might not correctly fit the clinician (Rees et al., 2021). Ill-fitting PPE is a health and safety risk; if clinicians are consciously aware of this, it is likely to reduce their confidence in its ability to protect them from contracting the virus and consequently impact their mental health (Hoernke et al., 2021; Rees et al., 2021; Vindrola-Padros et al., 2020). In this study, we observed average GHQ-12 scores decreasing through the three phases. Low confidence in using PPE could be a factor in why individual GHQ-12 scores were observed to increase. Healthcare organisations must ensure their staff can work safely to care for their patients, and it can be a source of psychological distress if adequate protection is unavailable to staff.

These findings were mirrored when participants were asked to rate their preparedness in treating patients with and without COVID-19. Participants who reported they were confident scored a lower GHQ-12 than those who were unconfident. Participant confidence is likely to be multi-faceted; as well as the issues with PPE, a lack of appropriate training has been identified as a factor likely to hinder a healthcare professional’s ability to perform their role effectively (Vindrola-Padros et al., 2020). Most CARA participants reported receiving formal training in using PPE and performing AGPs (Supplementary 3).

Effective communication ensures clinicians are prepared to undertake their clinical duties. Rees et al. (2021) interviewed 20 paramedics in one UK ambulance service, and reported that paramedics responded well to daily updates from their employer and virtual communication platforms such as Zoom. However, our findings suggest infrequently accessing employer guidance was associated with a lower GHQ-12 score. Although our study does not explain why GHQ-12 scores are lower when staff access Trust information infrequently, the frequency of accessing information could be a surrogate marker for psychological distress. Overall, employers should be cognisant of their means of communication and how engaged staff are with their messaging, as this is likely linked to well-being.

Most participants reported that they felt their health was at risk due to their clinical role, while over half reported no change; a proportion reported their health got worse. The health and well-being of ambulance paramedics have seen considerable attention in recent years (Gayton & Lovell, 2012; Meadley et al., 2020), reflecting both the physical and psychological risks to those in this occupation. Gayton and Lovell (2012) suggest that paramedics who report better health and well-being are more resilient to the occupational stresses encountered. Participants with pre-existing physical or mental health conditions had higher GHQ-12 scores and were worried that their pre-existing conditions might worsen due to being exposed to COVID-19. However, during phases 2 and 3, it was reported that participants’ mental health deteriorated, not their physical health. The study cannot fully elucidate the reasons behind the deterioration of mental health observed throughout the research period. However, it is likely attributable to a complex interplay of various factors. For instance, the anxiety induced in clinicians by the perceived risk of COVID-19 has been highlighted as a significant contributor (Rees et al., 2021). Additionally, the continuous media coverage has been reported to have an overwhelming and detrimental impact on individuals in the general population with pre-existing mental health conditions (Burton et al., 2023). The media’s fluctuating narrative on health services, such as the varying perspectives on remote consulting, has also played a role in this scenario (Mroz et al., 2021). Furthermore, the divisive social discourse surrounding the handling of the COVID-19 pandemic has added another layer of stress (Sanders et al., 2021).

Although mean GHQ-12 scores decreased over the study period, they were still higher than the threshold of 12, denoting a risk of psychological distress (Goldberg & Williams, 1988). This downward trend was also reported in the general population (Davillas & Jones, 2021) and in doctors in the ED (Roberts et al., 2021). There will likely be an element of adaption as society comes to terms with the new reality that COVID-19 has brought. Physical and psychological stress is not uncommon in the paramedic profession, and a range of coping strategies have been reported to mitigate the pressures on ambulance clinicians. These coping strategies can be formal, such as accessing occupational health support through their employer, and informal, such as peer support groups, family and friends (Barrett, 2016; Clompus & Albarran, 2016; Gayton & Lovell, 2012). Support packages and interventions are needed to ensure occupational stresses are mitigated appropriately. However, evidence to support the effectiveness of existing interventions for ambulance staff is absent (Clark et al., 2021). Roberts et al. (2021) commented that the support given to ED doctors is multi-level, accommodating the individual needs of staff and the organisation. Crucially, systems need to be in place to measure stressors, and evidence-based policies to ensure that workplace stressors do not harm staff.

### Limitations

This study has several limitations. Firstly, there are no baseline data regarding GHQ-12 scores in paramedics or the broader workforce within UK NHS ambulance trusts before COVID-19. Therefore, it is unclear whether the score reported in this study falls within the norm for this group. As suggested in the findings from this study, GHQ-12 scores fluctuate and provide only a snapshot of participants’ psychological stress when surveyed. Secondly, the studied population is large, including over 31,000 paramedics and an unknown number of non-registrant healthcare professionals in the UK ambulance setting. Given the study design, we cannot report on response rates, but it is clear that only a small proportion of the workforce was represented in this study. Whether the participants in this study represent the ‘average’ clinician is unknown. We acknowledge the possibility that staff affected by the COVID-19 pandemic were more inclined to share their views; furthermore, we did not undertake a subgroup analysis of paramedics working in clinical settings other than the ambulance service, due to the small number of respondents from the non-ambulance clinical setting, and therefore whether different clinical settings were a contributing factor to GHQ-12 scores. Thirdly, the survey took a snapshot of participants in three phases, and the results reflect how the study participants were when they completed the survey; while mental health was found to deteriorate over the three phases, it is unknown what the long-term effects were on the participants’ physical and mental health. Finally, only 58% of participants who initially agreed to participate completed all three phases of this study. Therefore, caution should be used when inferring changes throughout the study and whether these are due to the response to COVID-19 or the change in participant representation.

## Conclusion

Our results indicate that a large proportion of the UK NHS ambulance workforce experienced psychological distress during the first wave of the COVID-19 pandemic. While these stresses were observed to decrease, confidence in PPE and pre-existing mental health disorders appeared to be a significant factor in worsening psychological distress. Although reasons for causing and perpetuating distress are multi-factorial, ambulance managers need to be aware of the risks to staff mental health and take action to mitigate these, especially when staff are under additional pressures.

## Acknowledgements

Thank you to GL Assessment for donating the use of GHQ-12 with no cost as their support to research on COVID-19. Thank you to the Trainee Emergency Research Network (TERN) for sharing their early work to assist the CARA team in relation to gaining timely research approvals.

## Author contributions

The study was conceived by JW and SN. All authors contributed to the design of the survey and when each phase of data collection occurred. JWB, CM, TF, NR, AR, SB, FB, RF, AMS and MB supported recruitment. AH was responsible for managing the survey and data. Data analysis was led by KBE and supported by JWB, AH and PO. JWB drafted the manuscript and all authors contributed to its development and revisions. JWB acts as the guarantor for this article.

## Conflict of interest

JW is head of research at the College of Paramedics and a previous editorial board member of the *BPJ*. GM is the editor-in-chief of the *BPJ*.

## Ethics

Health Research Approval was granted on 30 March 2020 (Ref: 20/HRA/1654).

## Funding

This study was funded by the College of Paramedics, a unique grant awarded in response to exceptional circumstances.

## References

[bibr_1] Barrett J. W. (2016). Fit to practise: Does more need to be done to improve the health and wellbeing of paramedics? *Journal of Paramedic Practice*, 8(10), 487–492.

[bibr_2] Berger E. Winkelmann J. Eckhardt H. Nimptsch U. Panteli D. Reichebner C. Rombey T., & Busse R. (2022). A country-level analysis comparing hospital capacity and utilisation during the first COVID-19 wave across Europe. *Health Policy*, 126(5), 373–381. 34924210 10.1016/j.healthpol.2021.11.009 PMC8632742

[bibr_3] Blanchard J. Li Y. Bentley S. K. Lall M. D. Messman A. M. Liu Y. T. Diercks D. B. Merritt-Recchia R. Sorge R. Warchol J. M. Greene C. Griffith J. Manfredi R. A., & McCarthy M. (2022). The perceived work environment and well-being: A survey of emergency health care workers during the COVID-19 pandemic. *Academic Emergency Medicine*, 29(7), 851–861. 35531649 10.1111/acem.14519 PMC9347760

[bibr_4] Burton A. McKinlay A. Aughterson H., & Fancourt D. (2023). Impact of the COVID-19 pandemic on the mental health and well-being of adults with mental health conditions in the UK: A qualitative interview study. *Journal of Mental Health*, 32(6), 1040–1047. 34323635 10.1080/09638237.2021.1952953 PMC12094262

[bibr_5] Chua S. E. Cheung V. Cheung C. McAlonan G. M. Wong J. W. S. Cheung E. P. T. Chan M. T. Y. Wong M. M. C. Tang S. W. Choy K. M. Wong M. K. Chu C. M., & Tsang K. W. T. (2004). Psychological effects of the SARS outbreak in Hong Kong on high-risk health care workers. *Canadian Journal of Psychiatry. Revue canadienne de psychiatrie*, 49(6), 391–393. 15283534 10.1177/070674370404900609

[bibr_6] Clark L. V. Fida R. Skinner J. Murdoch J. Rees N. Williams J. Foster T., & Sanderson K. (2021). Mental health, well-being and support interventions for UK ambulance services staff: An evidence map, 2000 to 2020. *British Paramedic Journal*, 5(4), 25–39. 34421373 10.29045/14784726.2021.3.5.4.25 PMC8341070

[bibr_7] Clompus S. R., & Albarran J. W. (2016). Exploring the nature of resilience in paramedic practice: A psycho-social study. *International Emergency Nursing*, 28, 1–7. 26706122 10.1016/j.ienj.2015.11.006

[bibr_8] Davillas A., & Jones A. M. (2021). The first wave of the COVID-19 pandemic and its impact on socioeconomic inequality in psychological distress in the UK. *Health Economics (United Kingdom)*, 30(7), 1668–1683. 10.1002/hec.4275 PMC8207020 33904203

[bibr_9] Eaton-Williams P. J., & Williams J. (2023). ‘See us as humans. Speak to us with respect. Listen to us.’ A qualitative study on UK ambulance staff requirements of leadership while working during the COVID-19 pandemic. *BMJ Leader*, 7(2), 102–107. 10.1136/leader-2022-000622 37200184

[bibr_10] Ebben R. H. A. Woensdregt T. Wielenga-Meijer E. Pelgrim T. de Lange A. Berben S. A. A., & Vloet L. C. M. (2023). The impact of COVID-19 on the mental health and well-being of ambulance care professionals: A rapid review. *PLOS One*, 18(7), e0287821. https://doi.org/10.1371/journal.pone.0287821. 37432937 10.1371/journal.pone.0287821 PMC10335670

[bibr_11] Gayton S. D., & Lovell G. P. (2012). Resilience in ambulance service paramedics and its relationships with well-being and general health. *Traumatology*, 18(1), 58–64.

[bibr_12] Goldberg D. P., & Hillier V. F. (1979). A scaled version of the general health questionnaire. *Psychological Medicine*, 9(1), 139–145. 424481 10.1017/s0033291700021644

[bibr_13] Goldberg D. P., & Williams P. (1988). *A user’s guide to the general health questionnaire*. NFER-Nelson.

[bibr_14] Hoernke K. Djellouli N. Andrews L. Lewis-Jackson S. Manby L. Martin S. Vanderslott S., & Vindrola-Padros C. (2021). Frontline healthcare workers’ experiences with personal protective equipment during the COVID-19 pandemic in the UK: A rapid qualitative appraisal. *BMJ Open*, 11(1), e046199. https://doi.org/10.1136/bmjopen-2020-046199. 10.1136/bmjopen-2020-046199 PMC7818840 33472794

[bibr_15] Lancee W. J. Maunder R. G., & Goldbloom D. S. (2008). Prevalence of psychiatric disorders among Toronto hospital workers one to two years after the SARS outbreak. *Psychiatric Services (Washington, D. C.)*, 59(1), 91–95. 18182545 10.1176/ps.2008.59.1.91 PMC2923654

[bibr_16] Lawn S. Roberts L. Willis E. Couzner L. Mohammadi L., & Goble E. (2020). The effects of emergency medical service work on the psychological, physical, and social well-being of ambulance personnel: A systematic review of qualitative research. *BMC Psychiatry*, 20(1), 348. https://doi.org/10.1186/s12888-020-02752-4. 32620092 10.1186/s12888-020-02752-4 PMC7332532

[bibr_17] McCabe R. Schmit N. Christen P. D’Aeth J. C. Løchen A. Rizmie D. Nayagam S. Miraldo M. Aylin P. Bottle A. Perez-Guzman P. N. Ghani A. C. Ferguson N. M. White P. J., & Hauck K. (2020). Adapting hospital capacity to meet changing demands during the COVID-19 pandemic. *BMC Medicine*, 18(1), 329. https://doi.org/10.1186/s12916-020-01781-w. 33066777 10.1186/s12916-020-01781-w PMC7565725

[bibr_18] McKee M. (2020). England’s PPE procurement failures must never happen again. *BMJ*, 370, m2858. https://doi.org/10.1136/bmj.m2858. 32680840 10.1136/bmj.m2858

[bibr_19] Meadley B. Caldwell J. Perraton L. Bonham M. Powell Wolkow A. Smith K. Williams B., & Bowles K.-A. (2020). The health and well-being of paramedics: A professional priority. *Occupational Medicine*, 70(3), 149–151. 32459853 10.1093/occmed/kqaa039

[bibr_20] Monteiro Fonseca S. Faria S. Cunha S. Silva M. Ramos M. J. Azevedo G. Campos R. Barbosa A. R., & Queirós C. (2021). Mental health patterns during COVID-19 in emergency medical services (EMS). *International Journal of Emergency Services*, 11(2), 193–206.

[bibr_21] Mroz G. Papoutsi C. Rushforth A., & Greenhalgh T. (2021). Changing media depictions of remote consulting in COVID-19: Analysis of UK newspapers. *British Journal of General Practice*, 71(702), e1–e9. https://doi.org/10.3399/BJGP.2020.0967. 10.3399/BJGP.2020.0967 PMC7759365 33318086

[bibr_22] Poon Y.-S. R. Lin Y. P. Griffiths P. Kwang Yong K. Seah B., & Ying Liaw S. (2022). A global overview of healthcare workers’ turnover intention amid COVID-19 pandemic: A systematic review with future directions. *Human Resources for Health*, 20(1), 70. https://doi.org/10.1186/s12960-022-00764-7. 36153534 10.1186/s12960-022-00764-7 PMC9509627

[bibr_23] Ranney M. L. Griffeth V., & Jha A. K. (2020). Critical supply shortages — The need for ventilators and personal protective equipment during the Covid-19 pandemic. *New England Journal of Medicine*, 382(18), e41. https://doi.org/10.1056/NEJMp2006141. 32212516 10.1056/NEJMp2006141

[bibr_24] Rees N. Smythe L. Hogan C., & Williams J. (2021). Paramedic experiences of providing care in Wales (UK) during the 2020 COVID-19 pandemic (PECC-19): A qualitative study using evolved grounded theory. *BMJ Open*, 11(6), e048677. https://doi.org/10.1136/bmjopen-2021-048677. 10.1136/bmjopen-2021-048677 PMC8212156 34140344

[bibr_25] Roberts T. Daniels J. Hulme W. Hirst R. Horner D. Lyttle M. D. Samuel K. Graham B. Reynard C. Barrett M. Foley J. Cronin J. Umana E. Vinagre J. Carlton E., on behalf of The Trainee Emergency Research Network (TERN), Paediatric Emergency Research in the UK and Ireland (PERUKI) Research and Audit Federation of Trainees (RAFT), Irish Trainee Emergency Research Network (ITERN and Trainee Research in Intensive Care (TRIC)). (2021). Psychological distress and trauma in doctors providing frontline care during the COVID-19 pandemic in the United Kingdom and Ireland: A prospective longitudinal survey cohort study. *BMJ Open*, 11(7). https://doi.org/10.1136/bmjopen-2021-049680. 10.1136/bmjopen-2021-049680 PMC8275363 34244282

[bibr_26] Sanders J. G. Tosi A. Obradovic S. Miligi I., & Delaney L. (2021). Lessons from the UK’s lockdown: Discourse on behavioural science in times of COVID-19. *Frontiers in Psychology*, 12, 647348. https://doi.org/10.3389/fpsyg.2021.647348. 34220617 10.3389/fpsyg.2021.647348 PMC8247580

[bibr_27] Tabah A. Ramanan M. Laupland K. B. Buetti N. Cortegiani A. Mellinghoff J. Conway Morris A. Camporota L. Zappella N. Elhadi M. Povoa P. Amrein K. Vidal G. Derde L. Bassetti M. Francois G. Ssi Yan Kai N. De Waele J. J., & PPE-SAFE Contributors. (2020). Personal protective equipment and intensive care unit healthcare worker safety in the COVID-19 era (PPE-SAFE): An international survey. *Journal of Critical Care*, 59, 70–75. 32570052 10.1016/j.jcrc.2020.06.005 PMC7293450

[bibr_28] Taylor C. Mattick K. Carrieri D. Cox A., & Maben J. (2022). “The WOW factors”: Comparing workforce organization and well-being for doctors, nurses, midwives and paramedics in England. *British Medical Bulletin*, 141(1), 60–79. 35262666 10.1093/bmb/ldac003 PMC8935611

[bibr_29] Verelst F. Kuylen E., & Beutels P. (2020). Indications for healthcare surge capacity in European countries facing an exponential increase in coronavirus disease (COVID-19) cases, March 2020. *Eurosurveillance*, 25(13), 2000323. https://doi.org/10.2807/1560-7917.ES.2020.25.13.2000323. 32265003 10.2807/1560-7917.ES.2020.25.13.2000323 PMC7140594

[bibr_30] Vindrola-Padros C. Andrews L. Dowrick A. Djellouli N. Fillmore H. Bautista Gonzalez E. Javadi D. Lewis-Jackson S. Manby L. Mitchinson L. Mulcahy Symmons S. Martin S. Regenold N. Robinson H. Sumray K. Singleton G. Syversen A. Vanderslott S., & Johnson G. (2020). Perceptions and experiences of healthcare workers during the COVID-19 pandemic in the UK. *BMJ Open*, 10(11), e040503. https://doi.org/10.1136/bmjopen-2020-040503. 10.1136/bmjopen-2020-040503 PMC7646318 33154060

[bibr_31] Vujanovic A. A. Lebeaut A., & Leonard S. (2021). Exploring the impact of the COVID-19 pandemic on the mental health of first responders. *Cognitive Behaviour Therapy*, 50(4), 320–335. 33595426 10.1080/16506073.2021.1874506

[bibr_32] Winkelmann J. Webb E. Williams G. A. Hernández-Quevedo C. Maier C. B., & Panteli D. (2022). European countries’ responses in ensuring sufficient physical infrastructure and workforce capacity during the first COVID-19 wave. *Health Policy*, 126(5), 362–372. 34311982 10.1016/j.healthpol.2021.06.015 PMC9187509

